# Standardized Perioperative Thrombosis Prevention in Neonatal Modified Blalock–Taussig Shunt Surgery: An Algorithm-Based Single-Center Case Series

**DOI:** 10.3390/children13060766

**Published:** 2026-05-31

**Authors:** Valentin Stroe, Lacramioara Eliza Chiperi, Horatiu Suciu, Marius Harpa, David Emanuel Anitei, Liliana Gozar

**Affiliations:** 1 Institution Organizing University Doctoral Studies, University of Medicine, Pharmacy, Science and Technology “George Emil Palade”, 540142 Targu Mures, Romania; stroe.valentin-ionut24@stud.umfst.ro (V.S.); anitei.emanuel-david.25@stud.umfst.ro (D.E.A.); 2 Emergency Institute of Cardiovascular Diseases and Transplant, 540136 Targu Mures, Romania; horatiu.suciu@umfst.ro (H.S.); marius.harpa@umfst.ro (M.H.); liliana.gozar@umfst.ro (L.G.); 3 Pediatrics Departament, University of Medicine and Pharmacy “Iuliu Hatieganu”, 400012 Cluj-Napoca, Romania; 4 Department of Surgery IV, University of Medicine, Pharmacy, Science and Technology “George Emil Palade”, 540142 Targu Mures, Romania

**Keywords:** neonatal cardiac surgery, systemic-to-pulmonary shunt, modified Blalock–Taussig shunt, early shunt thrombosis, perioperative anticoagulation, thrombosis prevention

## Abstract

**Background/Objectives**: Early thrombosis of systemic-to-pulmonary artery shunts (SPS) remains a major cause of morbidity and mortality in neonates with duct-dependent pulmonary circulation. Despite advances in surgical technique, no universally accepted perioperative thrombosis-prevention protocol exists. We evaluated the early outcomes of a standardized perioperative thrombosis-prevention protocol applied in neonates undergoing SPS placement. **Methods**: This single-center case series included nine consecutive neonates undergoing primary modified Blalock–Taussig shunt placement between January 2024 and July 2025. A predefined and standardized perioperative thrombosis-prevention protocol was uniformly applied, incorporating preoperative aspirin when feasible, intraoperative systemic heparinization targeting activated clotting time (ACT) > 300 s, meticulous shunt flushing and de-airing, preferential distal anastomosis to the main pulmonary artery when anatomically suitable, and early postoperative continuous heparin infusion followed by enteral aspirin. The primary endpoint was early shunt thrombosis within 30 days. **Results**: Median age at surgery was 28 days (range 14–35), and median operative weight was 3.2 kg (range 2.8–3.6). Cardiopulmonary bypass was required in 33.3% of patients. Delayed sternal closure was performed in 22.2%. Despite recognized prothrombotic risk factors—including complex anatomy, hypoplastic pulmonary arteries, and low cardiac output syndrome (33.3%)—no early shunt thrombosis occurred (0/9). There were no reinterventions, no early mortality, and no major bleeding or intracranial hemorrhage. **Conclusions**: In this single-center neonatal series, implementation of a standardized perioperative thrombosis-prevention protocol was associated with preserved early shunt patency without increased bleeding risk. Although limited by a small sample size, these findings support the feasibility and short-term safety of a standardized perioperative management strategy in neonatal systemic-to-pulmonary shunt surgery. These findings should be considered hypothesis-generating and not evidence of definitive effectiveness.

## 1. Introduction

Systemic-to-pulmonary artery shunts (SPS) remain a cornerstone of palliative management in neonates with duct-dependent pulmonary circulation and complex congenital heart defects [1]. Despite technical refinements, acute and subacute shunt thrombosis continues to represent one of the most feared complications, frequently leading to sudden hypoxemia, hemodynamic collapse, and increased early mortality [2,3]. The incidence of shunt thrombosis in neonates is reported to be higher than in older infants, largely due to small vessel caliber, low-flow states, hematologic immaturity, and a prothrombotic inflammatory response following cardiothoracic surgery [2,4].

Multiple perioperative strategies have been proposed to mitigate the risk of shunt thrombosis, targeting surgical technique, anticoagulation protocols, and postoperative hemodynamic management [3,4,5].

Preoperative and early postoperative antiplatelet therapy, particularly aspirin administration, has been suggested as a preventive measure aimed at reducing early platelet aggregation and thrombus formation within the graft [5,6]. Intraoperatively, the use of systemic heparin prior to shunt construction is standard practice; some centers advocate higher activated clotting time (ACT) targets to reduce early thrombotic risk during vascular clamping and anastomosis [3,7].

Technical considerations may also influence thrombosis risk. Shunt configuration, anastomotic site selection, and optimization of shunt diameter relative to patient weight are recognized determinants of flow dynamics and patency [8,9]. Performing the distal anastomosis to the main pulmonary artery trunk rather than to a branch pulmonary artery may theoretically enhance flow distribution and reduce turbulence or competitive flow, although data remain limited [8]. Careful de-airing and flushing of the graft before full reperfusion is recommended to prevent early thrombotic occlusion secondary to blood stasis and endothelial injury [3].

In the immediate postoperative period, maintenance of an open sternum in selected high-risk neonates has been employed to reduce mediastinal compression, optimize cardiac output, and prevent low-flow states predisposing to shunt thrombosis [10]. Strict hemodynamic management aimed at avoiding hypotension, low cardiac output syndrome, hemoconcentration, and dehydration is critical in maintaining adequate shunt flow [3,4].

Additional strategies described in the literature include early postoperative continuous heparin infusion followed by transition to antiplatelet therapy, careful hematocrit control to avoid hyperviscosity, and vigilant monitoring for early signs of shunt dysfunction [4,6].

Although antiplatelet therapy, systemic heparinization, meticulous graft handling, and postoperative hemodynamic optimization are individually well established [4,5,8,10], their application in neonatal systemic-to-pulmonary shunt surgery remains highly heterogeneous across institutions. Published reports frequently describe single technical or pharmacologic measures in isolation, without embedding them within a clearly defined and reproducible perioperative framework [4,5,8,10]. In a field characterized by fragile physiology, narrow therapeutic margins, and substantial inter-center variability, fragmentation of care may itself represent a modifiable risk factor. The present study therefore focuses not on introducing a novel drug or surgical modification, but on evaluating a standardized, algorithm-based, and uniformly implemented perioperative pathway designed to integrate surgical precision, anticoagulation strategy, and physiologic management into a cohesive thrombosis-prevention model.

In this case series, we present nine neonatal patients undergoing systemic-to-pulmonary artery shunt placement in whom a standardized perioperative protocol incorporating selected thrombosis-prevention strategies was implemented. We analyze clinical characteristics, surgical techniques, anticoagulation management, and early outcomes, with particular focus on the incidence of shunt thrombosis and related complications.

## 2. Materials and Methods

### 2.1. Study Design and Patient Population

This case series included nine consecutive neonates who underwent systemic-to-pulmonary artery shunt placement at our institution between January 2024 and July 2025. All patients had duct-dependent pulmonary circulation secondary to complex congenital heart disease and were deemed candidates for palliative shunt surgery following multidisciplinary evaluation. Our institution functions as a tertiary referral center for complex congenital heart disease. All neonates meeting eligibility criteria during the study period were included consecutively. No additional eligible patients undergoing primary systemic-to-pulmonary artery shunt placement were excluded other than cases in which complete perioperative data were unavailable or in which the standardized perioperative protocol could not be fully implemented.

Inclusion criteria were: age ≤ 45 days at the time of surgery; primary systemic-to-pulmonary artery shunt procedure; availability of complete perioperative and follow-up data. Patients in whom the standardized protocol could not be followed were excluded.

The primary objective of this study was to evaluate the impact of a standardized perioperative thrombosis-prevention protocol on early shunt patency and thrombotic complications.

### 2.2. Perioperative Thrombosis-Prevention Protocol

A standardized protocol aimed at reducing shunt thrombosis was implemented and applied to all patients.

#### 2.2.1. Preoperative Management

When hemodynamically feasible and in the absence of contraindications (e.g., active bleeding, severe thrombocytopenia), aspirin was administered preoperatively at a dose of 3–5 mg/kg/day starting 24–48 h prior to surgery. Baseline laboratory evaluation included complete blood count, coagulation profile, fibrinogen, and assessment of hematocrit levels.

#### 2.2.2. Intraoperative Management

All procedures were performed via median sternotomy under general anesthesia and were undertaken by the same surgeon with the same surgical technique.

##### Anticoagulation Strategy

Systemic heparin was administered prior to vascular clamping at a dose of 150–200 IU/kg, targeting an ACT > 300 s before shunt construction. ACT was reassessed intraoperatively and heparin was supplemented as necessary.

##### Shunt Configuration and Anastomotic Technique

A polytetrafluoroethylene (PTFE) graft was used in all cases. The size of the SPS was selected according to body weight and pulmonary artery dimensions. A shorter graft length was used in order to prevent kinking of the graft and distortion of pulmonary vessels.

Whenever anatomically feasible, the distal anastomosis was performed to the main pulmonary artery trunk rather than to the right pulmonary artery branch, with the aim of promoting balanced pulmonary blood flow and minimizing localized turbulence.

The proximal anastomosis was created to the innominate artery. After completion of the proximal anastomosis and prior to full restoration of systemic flow, the shunt was carefully flushed (purged) to eliminate air and stagnant blood, thereby reducing the risk of early thrombus formation.

Flow adequacy was assessed by direct visualization, pressure monitoring, and intraoperative oxygen saturation response.

#### 2.2.3. Immediate Postoperative Management

In selected high-risk neonates (e.g., hemodynamic instability, myocardial edema, high inotropic support), delayed sternal closure was performed to reduce mediastinal compression and optimize cardiac output.

Continuous intravenous unfractionated heparin infusion was initiated within the first 6 h after surgery in the absence of active bleeding. In the majority of cases, anticoagulation was started between 2 and 4 h postoperatively once hemostasis was confirmed, and chest tube output was considered acceptable by the surgical team. It was started at 10–20 IU/kg/hour and titrated to maintain activated partial thromboplastin time (aPTT) within the institutional target range of approximately 60–80 s.

Anti-factor Xa monitoring was not routinely performed in this cohort. Intravenous unfractionated heparin was maintained until removal of mediastinal and pleural drainage tubes, allowing controlled anticoagulation during the period of highest bleeding risk. Following drain removal and confirmation of hemostatic stability, patients were transitioned to subcutaneous low-molecular-weight heparin to provide more stable anticoagulation during ongoing recovery. Enteral aspirin (3–5 mg/kg/day) was initiated or resumed once gastrointestinal function was established and clinical stability was achieved.

Hemodynamic management aimed to avoid systemic hypotension, prevent low cardiac output syndrome, maintain adequate preload, avoid dehydration and maintain hematocrit between 40–45%. Mechanical ventilation and vasoactive support were adjusted to prevent excessive pulmonary overcirculation and systemic hypoperfusion.

**Figure 1:**
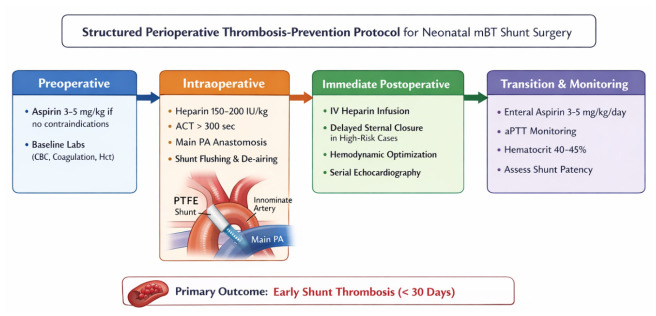
Algorithmic overview of the standardized perioperative thrombosis-prevention protocol implemented in neonates undergoing modified Blalock–Taussig shunt placement in this single-center case series. The protocol comprises four sequential phases: (1) preoperative evaluation with consideration of antiplatelet therapy and baseline hematologic assessment; (2) intraoperative systemic heparinization targeting activated clotting time (ACT) > 300 s, preferential distal anastomosis to the main pulmonary artery when anatomically feasible, and meticulous graft flushing and de-airing; (3) early postoperative intravenous unfractionated heparin infusion combined with hemodynamic optimization; and (4) transition to enteral aspirin therapy with continued coagulation monitoring and serial echocardiographic assessment of shunt patency. Abbreviations: ACT, activated clotting time; aPTT, activated partial thromboplastin time; PA, pulmonary artery; PTFE, polytetrafluoroethylene.

The standardized perioperative thromboprophylaxis protocol applied uniformly to all patients is summarized schematically in Figure 1 to facilitate reproducibility and transparency of the applied management strategy.

### 2.3. Data Collection

The following variables were recorded: demographic characteristics (gestational age, birth weight, weight at the time of surgery, and sex), underlying cardiac diagnosis, shunt size and configuration, and site of anastomosis. Intraoperative data included ACT values and total administered heparin dose. Postoperative variables comprised details of anticoagulation therapy, requirement for delayed sternal closure, inotropic support requirements, duration of mechanical ventilation, and length of stay in the intensive care unit (ICU) and in hospital.

### 2.4. Study Endpoints

The primary endpoint was early shunt thrombosis, defined as clinical and/or echocardiographic evidence of shunt occlusion requiring intervention or resulting in death within 30 days after surgery.

Secondary endpoints included reintervention for shunt dysfunction, early mortality (within 30 days), major bleeding complications, need for extracorporeal life support, and thromboembolic events.

Shunt patency was assessed by transthoracic echocardiography in conjunction with clinical evaluation. Screening for intracranial hemorrhage was routinely performed using cranial ultrasonography during the early postoperative period, according to institutional neonatal intensive care protocols.

### 2.5. Statistical Analysis

Given the exploratory nature of this case series and the limited sample size (n = 9), analyses were primarily descriptive.

Continuous variables were assessed for distribution using the Shapiro–Wilk test and are presented as median (range) due to non-normal distribution. Categorical variables are expressed as frequencies and percentages.

For the primary endpoint (early shunt thrombosis), the exact binomial 95% confidence interval was calculated to contextualize the observed event rate.

Given the absence of a control group and the limited sample size, no inferential comparative statistical testing was performed. The study was not powered to detect differences in thrombosis incidence; therefore, findings are interpreted descriptively and considered hypothesis-generating.

Statistical analysis was performed using GraphPad InStat version 3.06 and Microsoft Excel Office 2021 software.

### 2.6. Ethical Considerations

The study was conducted in accordance with the Declaration of Helsinki. Institutional Review Board approval was obtained (Approval No. 1442). Informed consent was obtained from parents or guardians of the patients.

## 3. Results

### 3.1. Study Population

Nine consecutive neonates with duct-dependent pulmonary circulation underwent modified Blalock–Taussig shunt placement during the study period. Given the limited sample size (n = 9), no formal statistical comparisons were performed, and findings are reported descriptively.

Median age at surgery was 28 days (range 14–35), and median operative weight was 3.2 kg (range 2.8–3.6). All patients had complex congenital heart disease with functionally univentricular physiology or severely compromised right ventricular outflow.

Diagnostic substrates included tricuspid atresia, single ventricle with pulmonary atresia, severe right ventricular hypoplasia/atresia, and extreme tetralogy of Fallot with pulmonary atresia. Associated anomalies were present in three patients (33.3%), including heterotaxy with dextrocardia (n = 1) and anomalous pulmonary venous drainage (n = 2). Hypoplastic pulmonary arteries were documented in all cases.

Preoperative prostaglandin E1 infusion was required in 100% of patients. Enteral Aspirin (dose of 3–5 mg/kg/day) was started in all patients before surgery.

Baseline characteristics of included patients are summarized in Table 1.

To better characterize the heterogeneity and anatomical complexity of the cohort, detailed individual cardiac diagnoses and associated anatomical features are summarized in Table 2.

### 3.2. Operative Characteristics

All procedures were performed via median sternotomy using a PTFE (Gore-Tex) graft by the same surgeon using a consistent surgical technique. Shunt diameter ranged from 3.5 to 4.0 mm, selected according to body weight and pulmonary artery dimensions.

Proximal anastomosis was constructed to the brachiocephalic or subclavian artery, and distal anastomosis to the main pulmonary artery.

Cardiopulmonary bypass was required in 3 patients (33.3%), primarily due to anatomical complexity or hemodynamic instability. The remaining procedures were completed off-pump.

All patients received systemic heparinization before vascular clamping. Standardized shunt flushing and meticulous de-airing were systematically performed.

No intraoperative shunt thrombosis occurred. One patient exhibited intraoperative coagulation instability with intrapericardial thrombus formation; however, direct inspection and intraoperative assessment confirmed preserved shunt patency. Operative data are detailed in Table 3.

### 3.3. Early Postoperative Course

A standardized antithrombotic protocol was applied in all cases. Early postoperative anticoagulation consisted of continuous intravenous unfractionated heparin infusion, initiated early after surgery, typically within the first hours following admission to the intensive care unit once adequate hemostasis had been confirmed and maintained until removal of drainage tubes. After confirmation of hemostatic stability and removal of drainage tubes, patients were transitioned to subcutaneous low-molecular-weight heparin. Enteral aspirin (3–5 mg/kg/day) was initiated after clinical stabilization and establishment of enteral tolerance.

Delayed sternal closure was required in 2 patients (22.2%) due to myocardial edema and hemodynamic instability.

All patients required postoperative mechanical ventilation. Median ventilation duration was 4 days (range 2–8). Median ICU stay was 9 days (range 6–15), and median total hospital stay was 22 days (range 16–32).

**Figure 2:**
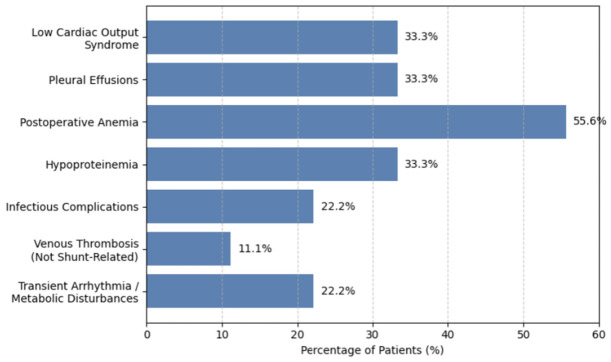
Postoperative complications in neonates after systemic-to-pulmonary shunt surgery.

Postoperative complications were consistent with the severity of underlying physiology and are reported in Figure 2. All complications were managed conservatively and resolved before discharge.

Patient-level operative characteristics, postoperative complications, and discharge outcomes are summarized in Table 4.

### 3.4. Primary Endpoint: Early Shunt Thrombosis

No early shunt thrombosis occurred within 30 days (0/9). Serial echocardiography demonstrated preserved shunt patency in all patients, with continuous systemic-to-pulmonary flow and no evidence of obstruction or flow acceleration suggestive of impending thrombosis.

Importantly, even in the presence of recognized prothrombotic and hemodynamic risk factors—including low cardiac output, ventricular dysfunction, delayed sternal closure, and intraoperative coagulation instability—no thrombotic events involving the systemic-to-pulmonary shunt were observed.

With zero events observed (0/9), the exact 95% confidence interval for early thrombosis was 0% to approximately 33%, reflecting the uncertainty inherent to a small sample size.

### 3.5. Secondary Endpoints

No patient required surgical or catheter-based reintervention for shunt dysfunction. Thirty-day mortality was 0%. Routine postoperative cranial ultrasonography did not identify intracranial hemorrhage in any patient. No major bleeding events, intracranial hemorrhage, or systemic arterial thromboembolic events were documented. Postoperative outcomes are summarized in Table 5.

One patient developed catheter-associated femoral venous thrombosis during the early postoperative period. The diagnosis was established by Doppler ultrasonography following localized lower-limb edema. The event occurred without associated shunt dysfunction or systemic embolic complications. Therapeutic anticoagulation was continued with low-molecular-weight heparin, with subsequent clinical improvement and no requirement for invasive intervention.

## 4. Discussion

Systemic-to-pulmonary artery shunt placement remains a life-saving palliative procedure in neonates with duct-dependent pulmonary circulation. Despite technical advances, shunt thrombosis continues to represent one of the most critical early postoperative complications, often presenting abruptly and carrying substantial mortality risk [11,12]. Neonates are particularly vulnerable due to small vessel diameter, low cardiac output states, hematologic immaturity, and a pronounced inflammatory and prothrombotic response following cardiopulmonary stress [13,14].

Reported rates of early systemic-to-pulmonary shunt thrombosis in neonates vary across studies but are generally described in the range of approximately 5–15% [2,5,15,16,17,18,19], with some series reporting even higher rates in high-risk anatomical or low-birth-weight populations. Although differences in patient selection, surgical technique, anticoagulation strategies, and definitions of thrombosis limit direct comparison, the absence of early shunt occlusion in our cohort may be considered clinically encouraging when interpreted cautiously in the context of published data. Nevertheless, given the small sample size and wide confidence interval associated with zero observed events, this observation should be interpreted cautiously and considered hypothesis-generating rather than confirmatory.

### 4.1. Interpretation of the Primary Finding

In this series of nine high-risk neonates, no early shunt thrombosis occurred within 30 days. While this finding is clinically encouraging, it must be interpreted within the context of the limited sample size. With zero observed events, the upper bound of the 95% confidence interval remains clinically relevant, underscoring the need for cautious interpretation despite the absence of thrombotic complications.

Importantly, this outcome was observed in a biologically vulnerable cohort characterized by complex univentricular physiology, hypoplastic pulmonary arteries, postoperative low cardiac output syndrome (33.3%), delayed sternal closure (22.2%), and intraoperative coagulation instability in one case. These features are well-recognized contributors to early shunt occlusion and would typically be associated with a non-negligible thrombotic risk.

Rather than suggesting definitive efficacy, the absence of early thrombosis in this context supports the plausibility that a standardized perioperative thrombosis-prevention protocol—combined with meticulous surgical technique and structured postoperative management—may be feasibly implemented during the vulnerable early postoperative period without an apparent increase in major bleeding complications. Accordingly, the present findings should be viewed as hypothesis-generating and supportive of protocol-driven management, warranting validation in larger, preferably multicenter cohorts.

Because no parallel control cohort receiving standard perioperative management was available, it is not possible to determine whether the observed outcomes were related to the implemented protocol itself, institutional surgical expertise, postoperative intensive care practices, or patient-related factors.

**Figure 3:**
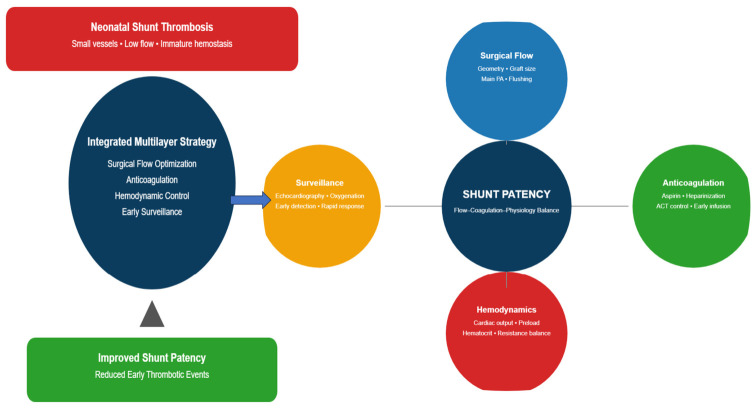
Integrated multilayer model for prevention of systemic-to-pulmonary shunt thrombosis in neonates. Shunt patency depends on the dynamic interaction between surgical flow optimization, structured anticoagulation, strict hemodynamic control, and vigilant postoperative surveillance. The model highlights the physiology-driven and interdependent determinants of thrombosis prevention in neonatal cardiac surgery.

Therefore, we propose a conceptual multilayer protection model integrating surgical, pharmacological, and physiological domains, reflecting the complex and interdependent determinants of shunt patency in neonates (Figure 3). The algorithmic structure of the protocol may facilitate adoption and reproducibility in other neonatal cardiac centers.

### 4.2. Mechanistic Considerations and Surgical Strategy

Shunt thrombosis in neonates is multifactorial. Contributing mechanisms include low-flow states, turbulent flow within small-diameter conduits, endothelial activation and inflammation, immature neonatal coagulation pathways, and hemoconcentration following cardiopulmonary stress [13,14].

Refinements in surgical strategy aimed at optimizing laminar flow and minimizing turbulence have substantially improved shunt reproducibility over recent decades. The modified Blalock–Taussig shunt using PTFE grafts represented a major technical advance compared to the classic configuration [20]. Shunt diameter selection tailored to body weight is critical, as oversized conduits may lead to pulmonary overcirculation and systemic hypoperfusion, whereas undersized shunts predispose to low-flow states and thrombosis [21]. A shorter graft length was used because in graft-based anastomosis, minimizing graft length is essential to prevent distortion of the adjacent vascular structures.

In our series, graft size selection and preferential distal anastomosis to the main pulmonary artery were individualized according to patient anatomy to optimize flow geometry. Although comparative data remain limited, attention to conduit configuration and shear stress patterns is increasingly recognized as an important determinant of patency.

The use of a main pulmonary artery–based anastomosis in the reconstruction strategy should also be interpreted cautiously. The present study was not designed to evaluate the superiority of a specific surgical configuration. Rather, the technique was applied as part of the institutional surgical approach. While this configuration may offer theoretical advantages related to flow dynamics and technical reproducibility, the current data do not allow conclusions regarding its superiority compared with alternative reconstructive strategies. Larger comparative studies would be required to clarify whether this aspect of the surgical technique has an independent impact on thrombosis risk or early outcomes.

Meticulous intraoperative graft flushing prior to reperfusion represents an additional preventive measure. Even brief periods of blood stasis within a small-caliber PTFE conduit may promote thrombus formation in neonates with reactive platelets and elevated fibrinogen levels [13]. Standardization of this maneuver may represent a simple yet clinically meaningful intervention within a structured protocol.

### 4.3. Antithrombotic Therapy: Between Evidence and Variability

Antithrombotic management in neonates remains characterized by institutional heterogeneity. Although low-dose aspirin following systemic-to-pulmonary shunt placement has been associated with improved patency in observational studies [15,22], high-quality neonatal-specific randomized data remain limited. Current pediatric antithrombotic guidelines support antiplatelet therapy in this setting [23], yet optimal dosing strategies and timing are not fully standardized.

Similarly, while intraoperative systemic heparinization is universally adopted, ACT targets and postoperative infusion protocols vary considerably among centers [24]. Developmental hemostasis in neonates further complicates management, as the balance between thrombosis and bleeding differs substantially from that in older children and adults [14].

Our standardized perioperative thrombosis-prevention protocol reflects an attempt to bridge this vulnerable early postoperative window by combining preoperative Aspirin administration, intraoperative ACT-guided anticoagulation, early postoperative unfractionated heparin infusion, and timely transition to aspirin. Although no major bleeding events were observed in this small cohort, conclusions regarding bleeding safety remain preliminary and require confirmation in larger studies.

### 4.4. Hemodynamic Optimization and Clinical Implications

Beyond surgical and pharmacologic strategies, postoperative hemodynamic optimization is central to thrombosis prevention. Low cardiac output syndrome, systemic hypotension, elevated hematocrit, and dehydration reduce shunt flow velocity and increase thrombogenicity [13,20]. The interplay between pulmonary and systemic vascular resistance further complicates management, as excessive pulmonary vasodilation may compromise systemic perfusion, whereas elevated pulmonary vascular resistance may limit shunt flow.

Delayed sternal closure, when indicated, may indirectly protect against shunt thrombosis by improving ventricular compliance and maintaining forward flow during the inflammatory phase [25]. In our cohort, delayed closure did not result in thrombotic complications when integrated within the standardized protocol.

These observations reinforce the concept that thrombosis prevention in neonatal modified Blalock–Taussig shunt surgery should be protocol-driven rather than reactive. As illustrated in Figure 1, the integration of surgical precision with predefined pharmacologic and hemodynamic targets supports a structured management approach that may mitigate early thrombotic risk in this fragile population.

### 4.5. Strengths and Limitations

This study has several strengths. All procedures were performed by the same surgeon using a consistent surgical technique, reducing technical variability. A predefined and standardized perioperative thrombosis-prevention protocol (presented in Figure 1) was uniformly applied to all patients, ensuring management consistency across the cohort. Detailed perioperative data—including anticoagulation timing, ACT targets, and postoperative hemodynamic parameters—were systematically collected. In addition, shunt patency was assessed through serial echocardiographic evaluation, enhancing endpoint reliability. Importantly, the cohort represents a biologically high-risk neonatal population, increasing the clinical relevance of the findings.

Several limitations must be acknowledged. This is a small single-center case series (n = 9), limiting statistical power and precluding comparative analysis. The relatively small sample size should be interpreted in the context of the rarity and complexity of neonates undergoing the SSP procedure. Even in high-volume congenital heart centers, the number of patients treated annually remains limited. The present study therefore reflects real-world experience within a specialized neonatal cardiac surgery program rather than a large registry analysis. Despite the modest cohort size, the uniformity of the perioperative management protocol provides valuable insight into the feasibility of standardized thrombosis prevention strategies in this highly vulnerable population.

Another limitation is the absence of a formal control group. The purpose of the present study was not to perform a comparative effectiveness analysis but rather to describe the outcomes associated with the implementation of a standardized perioperative protocol within a single institutional practice. Future multicenter studies comparing ductal stenting, different surgical techniques and anticoagulation strategies may help clarify the relative contribution of specific components of perioperative management to thrombosis prevention.

An additional limitation is the bundled nature of the intervention strategy. The implemented protocol incorporated multiple interdependent components, including preoperative aspirin administration, intraoperative systemic heparinization, graft handling techniques, postoperative anticoagulation, and strict hemodynamic optimization. Consequently, the present study cannot determine the relative contribution of any individual component to the observed outcomes. The findings therefore reflect the feasibility of an integrated perioperative pathway rather than the efficacy of a specific isolated intervention.

Furthermore, anticoagulation monitoring was performed using aPTT without routine anti-factor Xa measurements, which may result in variability in the assessment of heparin activity. Although systematic cerebral imaging screening was performed to identify intracranial hemorrhage, subclinical neurological events cannot be entirely excluded.

The study also focuses primarily on early postoperative outcomes. Our objective was specifically to evaluate thrombotic events occurring in the immediate postoperative window, when the risk of shunt or conduit thrombosis is highest. For this reason, the analysis was limited to early postoperative outcomes rather than medium- or long-term follow-up. Although no early thrombosis was observed, the upper bound of the 95% confidence interval remains clinically relevant, emphasizing the exploratory nature of these findings. Longitudinal evaluation of neurodevelopmental outcomes, late thrombosis, and interstage complications represents an important area for future investigation and will require extended follow-up of this patient cohort.

Taken together, the findings of the present study suggest that in neonatal cardiac surgery, where physiological reserve is extremely limited, consistent application of a structured perioperative pathway may represent an important strategy for reducing variability in postoperative management. Accordingly, the results should be interpreted as hypothesis-generating and supportive of further prospective validation. Further multicenter studies will be required to validate these observations and to determine whether standardized protocols can contribute to improved outcomes in this complex patient population.

### 4.6. Future Directions

Despite major advances in neonatal cardiac surgery—including improvements in perfusion strategies, anesthesia, and intensive care—systemic-to-pulmonary shunt thrombosis remains emblematic of the broader challenges of neonatal physiology: small anatomy, immature hemostasis, and narrow therapeutic margins [12]. The absence of universally accepted thrombosis-prevention protocols reflects limited prospective neonatal data and ongoing institutional variability.

Prospective multicenter studies incorporating standardized anticoagulation pathways and systematic outcome reporting are needed to define optimal strategies, balance thrombosis prevention against bleeding risk, and identify patient-specific predictors of shunt occlusion.

### 4.7. Early Thrombosis After Systemic-to-Pulmonary Shunts: Literature Comparison

To contextualize our findings within the current evidence base, we compared reported early thrombosis rates and perioperative antithrombotic strategies from key published cohorts involving neonates and infants with systemic-to-pulmonary artery shunts.

Early thrombosis rate generally ranges between 5% and 15%, depending on study design, patient selection, and thrombosis definitions [2,5,16,17,18,19,22,26,27,28].

A recent systematic review and meta-analysis encompassing nearly 5000 patients reported a pooled early shunt thrombosis rate of 8.4%, with variability across different anticoagulant/antiplatelet regimens, highlighting the heterogeneity of practice and outcomes in this population [17]. Smaller cohorts with protocolized strategies, such as immediate postoperative bivalirudin followed by low-molecular-weight heparin and aspirin, have demonstrated zero early thrombosis events in limited samples, though larger confirmatory studies are lacking [18]. Historically reported retrospective cohorts indicate early thrombosis or shunt failure rates in the high single-digit range, and the CLARINET randomized trial in infants did not show a benefit of clopidogrel added to aspirin, with a high overall rate of the composite thrombotic endpoint [16]. These comparisons underscore the persistent clinical challenge of shunt thrombosis and the need for standardized perioperative protocols.

Importantly, substantial variability exists in perioperative antithrombotic regimens, with aspirin, unfractionated heparin, and combination approaches used inconsistently across institutions. Although direct comparison is limited by heterogeneity in definitions and follow-up duration, the absence of early shunt thrombosis in our cohort may be viewed as clinically encouraging, although interpretation remains limited by the exploratory nature of the study. However, given the small sample size and wide confidence interval associated with zero observed events, this finding should be interpreted cautiously and considered hypothesis-generating rather than definitive evidence of superiority.

Across published studies, substantial variability persists regarding postoperative anticoagulation regimens, monitoring strategies, and timing of antiplatelet therapy initiation. In our cohort, early postoperative intravenous heparin monitored by aPTT, combined with systematic echocardiographic surveillance and routine cranial ultrasonography screening, reflected an effort to balance thrombosis prevention against bleeding risk within a structured perioperative framework.

### 4.8. Final Points

The contribution of this study lies in the operationalization of established thrombosis-prevention principles into a standardized, reproducible perioperative pathway tailored to neonatal physiology. Rather than proposing isolated anticoagulation regimens or surgical technical refinements, we describe an integrated management algorithm that aligns intraoperative anticoagulation targets, graft handling strategy, early postoperative heparinization, and strict hemodynamic optimization within a single structured framework. We sought to reduce variability during the vulnerable early postoperative window by this approach.

Although the present findings are limited by sample size and observational design, they support the feasibility of implementing an integrated, protocolized approach and highlight the potential value of reproducible perioperative pathways in neonatal cardiac surgery.

In the context of ongoing variability in neonatal cardiac surgical practice, such protocol-driven standardization may represent a potentially useful framework for reducing variability in perioperative management. While confirmatory data from larger, multicenter studies are required, our findings support the feasibility and potential value of integrated perioperative pathways in supporting standardized perioperative care in neonatal systemic-to-pulmonary shunt surgery.

In neonatal cardiac surgery, where physiological margins are narrow, disciplined reduction in variability through structured perioperative pathways may itself constitute a meaningful step toward improving early outcomes.

## 5. Conclusions

In this single-center series of neonates undergoing modified Blalock–Taussig shunt placement for duct-dependent pulmonary circulation, no early shunt thrombosis was observed despite significant anatomic and hemodynamic risk factors.

Implementation of a standardized perioperative thrombosis-prevention protocol appeared feasible and was not associated with increased major bleeding during the early postoperative period. However, given the small sample size, absence of a comparator group, and restriction to short-term follow-up, the present findings should be interpreted cautiously and considered hypothesis-generating only. The durability of shunt patency, interstage outcomes, and long-term thrombotic risk remain unknown and require further evaluation in larger prospective studies.

## Figures and Tables

**Table 1:** Baseline demographic and preoperative characteristics.

Variable	Value
Number of patients	9
Sex	6 male, 3 female
Gestational age	36–40 weeks
Birth weight, kg	2.2–3.4
Weight at surgery, kg	2.8–3.6
Diagnosis	Single ventricle physiology, pulmonary/tricuspid atresia, extreme TOF
Duct-dependent circulation	9 (100%)
Preoperative PGE1	9 (100%)
Preoperative hematologic abnormalities	5 (55.6%)

**Table 2:** Detailed cardiac anatomy and associated features of the study cohort.

Patient	Primary Cardiac Diagnosis	Associated Anatomical Features
**Patient 1**	Tricuspid atresia with normally related great arteries and pulmonary atresia	Severe right ventricular hypoplasia; hypoplastic main and left pulmonary arteries
**Patient 2**	Pulmonary atresia with duct-dependent pulmonary circulation	Ventricular inversion; unrestricted atrial and ventricular septal defects; reverse-angle patent ductus arteriosus
**Patient 3**	Functionally univentricular heart (right ventricular type)	Unbalanced atrioventricular canal defect; double-outlet right ventricle with malposed great arteries; severe subvalvular and valvular pulmonary stenosis; hypoplastic pulmonary annulus and pulmonary arteries; persistent left superior vena cava; dextrocardia; thoracic situs inversus
**Patient 4**	Pulmonary atresia with anatomically corrected transposition of the great arteries	Large outlet ventricular septal defect; large atrial septal defect; hypoplastic pulmonary trunk and branches; dextrocardia
**Patient 5**	Pulmonary atresia with intact ventricular septum	Bipartite hypoplastic right ventricle; dysplastic tricuspid valve with moderate-to-severe stenosis and regurgitation; aneurysmal atrial septum
**Patient 6**	Functionally univentricular heart (left ventricular type) with pulmonary atresia	Double-inlet left ventricle; severe hypoplasia/subat-resia of the right atrioventricular valve; hypoplastic pulmonary trunk; heterotaxy syndrome; interrupted inferior vena cava; dextrocardia
**Patient 7**	Tricuspid atresia with normally related great arteries	Nonrestrictive bulboventricular foramen; hypoplastic pulmonary annulus and pulmonary arteries; aneurysmal atrial septum
**Patient 8**	Complex cyanotic congenital heart disease with single-ventricle physiology (left ventricular type)	Tricuspid atresia; severe right ventricular hypoplasia; pulmonary atresia; confluent pulmonary arteries; dextrocardia; thoracic situs inversus
**Patient 9**	Extreme tetralogy of Fallot with pulmonary atresia	Rudimentary pulmonary trunk; hypoplastic confluent pulmonary arteries; small ostium secundum atrial septal defect

Abbreviations: ASD, atrial septal defect; VSD, ventricular septal defect.

**Table 3:** Intraoperative characteristics.

Variable	Value
Surgical approach	Median sternotomy
Shunt type	Modified Blalock–Taussig
Graft material	PTFE (Gore-Tex)
Shunt diameter, mm	3.5–4
Proximal anastomosis	Brachiocephalic/Subclavian artery
Distal anastomosis	Pulmonary artery trunk
Cardiopulmonary bypass	Yes (3), No (6)
Shunt flushing and de-airing	9 (100%)
Intraoperative shunt thrombosis	0

**Table 4:** Patient-level operative characteristics, postoperative complications, and discharge outcomes.

Patient	Diagnosis Category	Shunt Size (mm)	CPB	Delayed Sternal Closure	Major Complications	Discharge Status
**Patient 1**	Tricuspid atresia with pulmonary atresia	3.5	No	No	None	Discharged
**Patient 2**	Pulmonary atresia with ventricular inversion	3.5	No	No	Catheter-associated femoral venous thrombosis	Discharged
**Patient 3**	Functionally univentricular heart (right ventricular type)	4.0	Yes	Yes	Low cardiac output syndrome	Discharged
**Patient 4**	Pulmonary atresia with corrected transposition of the great arteries	3.5	No	No	None	Discharged
**Patient 5**	Pulmonary atresia with intact ventricular septum	3.5	No	No	Ventricular dysfunction	Discharged
**Patient 6**	Functionally univentricular heart (left ventricular type) with pulmonary atresia	4.0	Yes	Yes	Low cardiac output syndrome	Discharged
**Patient 7**	Tricuspid atresia with normally related great arteries	3.5	No	No	None	Discharged
**Patient 8**	Complex cyanotic congenital heart disease with single-ventricle physiology	4.0	Yes	No	Intraoperative coagulation instability	Discharged
**Patient 9**	Extreme tetralogy of Fallot with pulmonary atresia	3.5	No	No	None	Discharged

Abbreviations: CPB, cardiopulmonary bypass.

**Table 5:** Postoperative outcomes.

Variable	Value
Postoperative heparin infusion	9 (100%)
Aspirin initiated	9 (100%)
Delayed sternal closure	2 (22.2%)
Mechanical ventilation, days	2–8
ICU stay, days	6–15
Early shunt thrombosis	0
Reintervention	0
Venous thrombosis (non-shunt)	1 (11.1%)
Early mortality (30 days)	0

## Data Availability

The data presented in this study are available upon request from the corresponding author. The data are not publicly available due to privacy and ethical reasons.

## Abbreviations

The following abbreviations are used in this manuscript:

SSP	Systemic-to-Pulmonary Shunt
ACT	Activated Clotting Time
aPTT	Activated Partial Thromboplastin Time
PA	Pulmonary artery
PTFE	Polytetrafluoroethylene
ICU	Intensive Care Unit

## References

[1] Blalock A., Taussig H.B. (1945). The surgical treatment of malformations of the heart. JAMA.

[2] Petrucci O., O’Brien S.M., Jacobs M.L., Jacobs J.P., Manning P.B., Eghtesady P. (2011). Risk Factors for Mortality and Morbidity after the Neonatal Blalock–Taussig Shunt Procedure. Ann. Thorac. Surg..

[3] Tweddell J.S., Hoffman G.M., Mussatto K.A., Fedderly R.T., Berger S., Jaquiss R.D., Ghanayem N.S., Frisbee S.J., Litwin S.B. (2002). Improved survival of patients undergoing palliation of hypoplastic left heart syndrome: Lessons learned from 115 consecutive patients. Circulation.

[4] Monagle P., Cochrane A., Roberts R., Manlhiot C., Weintraub R., Szechtman B., Hughes M., Andrew M., McCrindle B.W. (2011). Fontan Anticoagulation Study Group. A multicenter, randomized trial comparing heparin/warfarin and acetylsalicylic acid as primary thromboprophylaxis for 2 years after the Fontan procedure in children. J. Am. Coll. Cardiol..

[5] Li J.S., Yow E., Berezny K.Y., Rhodes J.F., Bokesch P.M., Charpie J.R., Forbus G.A., Mahony L., Boshkov L., Lambert V. (2007). Clinical outcomes of palliative surgery including a systemic-to-pulmonary artery shunt in infants with cyanotic congenital heart disease: Does aspirin make a difference?. Circulation.

[6] Monagle P., Chan A.K.C., Goldenberg N.A., Ichord R.N., Journeycake J.M., Nowak-Göttl U., Vesely S.K. (2012). Antithrombotic therapy in neonates and children: Antithrombotic Therapy and Prevention of Thrombosis, 9th ed: American College of Chest Physicians Evidence-Based Clinical Practice Guidelines. Chest.

[7] Guzzetta N.A., Faraoni D., Josephson C.D. (2023). Hemostasis Management of the Pediatric Surgical Patient.

[8] Shiraishi S., Watanabe M., Sugimoto A., Tsuchida M. (2024). Surgical outcomes of the systemic-to-pulmonary artery shunt: Risk factors of post-operative acute events and effectiveness of regulation of pulmonary blood flow with metal clips. Gen. Thorac. Cardiovasc. Surg..

[9] de Leval M.R. (2004). Comprehensive Surgical Management of Congenital Heart Disease. J. R. Soc. Med..

[10] Elassal A.A., Eldib O.S., Dohain A.M., Abdelmohsen G.A., Abdalla A.H., Al-Radi O.O. (2019). Delayed Sternal Closure in Congenital Heart Surgery: A Risk-Benefit Analysis. Heart Surg. Forum.

[11] McMullan D.M., Permut L.C., Jones T.K., Johnston T.A., Rubio A.E. (2014). Modified Blalock-Taussig shunt versus ductal stenting for palliation of cardiac lesions with inadequate pulmonary blood flow. J. Thorac. Cardiovasc. Surg..

[12] Alkhulaifi A.M., Lacour-Gayet F., Serraf A., Belli E., Planché C. (2000). Systemic pulmonary shunts in neonates: Early clinical outcome and choice of surgical approach. Ann. Thorac. Surg..

[13] De Oliveira N.C., Ashburn D.A., Khalid F., Burkhart H.M., Adatia I.T., Holtby H.M., Williams W.G., Van Arsdell G.S. (2004). Prevention of early sudden circulatory collapse after the Norwood operation. Circulation.

[14] Monagle P., Ignjatovic V., Savoia H. (2010). Hemostasis in neonates and children: Pitfalls and dilemmas. Blood Rev..

[15] Manlhiot C., Brandão L.R., Kwok J., Kegel S., Menjak I.B., Carew C.L., Chan A.K., Schwartz S.M., Sivarajan V.B., Caldarone C.A. (2012). Thrombotic complications and thromboprophylaxis across all three stages of single ventricle heart palliation. J. Pediatr..

[16] Wessel D.L., Berger F., Li J.S., Dähnert I., Rakhit A. (2013). Clopidogrel in Infants with Systemic-to-Pulmonary-Artery Shunts. N. Engl. J. Med..

[17] Kiskaddon A.L., Goldenberg N.A., Betensky M., Branstetter J.W., Ashour D., Williams P., Stock A.C., Silvey M., Giglia T.M., Do N.L. (2025). Antithrombotic Strategies and Outcomes in Neonates and Infants with Cardiac Shunts: A Systematic Review and Meta-Analysis. Res. Pract. Thromb. Haemost..

[18] Soderstrom R.J., Chernuta E.C., Chaudhry-Waterman N., Rafter J.A., Stone M.L., Kim J.S. (2025). Successful use of bivalirudin in neonates for postoperative aortopulmonary shunt thrombosis prevention. JTCVS Tech..

[19] Giglia T.M., Massicotte M.P., Tweddell J.S., Barst R.J., Bauman M., Erickson C.C., Feltes T.F., Foster E., Hinoki K., Ichord R.N. (2013). Prevention and treatment of thrombosis in pediatric and congenital heart disease: A scientific statement from the American Heart Association. Circulation.

[20] de Leval M.R., McKay R., Jones M., Stark J., Macartney F.J. (1981). Modified Blalock-Taussig shunt. Use of subclavian artery orifice as flow regulator in prosthetic systemic-pulmonary artery shunts. J. Thorac. Cardiovasc. Surg..

[21] Jonas R.A. (2014). Comprehensive Surgical Management of Congenital Heart Disease.

[22] Tweddell J.S. (2007). Aspirin: A treatment for the headache of shunt-dependent pulmonary blood flow and parallel circulation?. Circulation.

[23] Douketis J.D., Spyropoulos A.C., Spencer F.A., Mayr M., Jaffer A.K., Eckman M.H., Dunn A.S., Kunz R. (2012). Perioperative management of antithrombotic therapy: Antithrombotic Therapy and Prevention of Thrombosis, 9th ed: American College of Chest Physicians Evidence-Based Clinical Practice Guidelines. Chest.

[24] Thom K.E., Hanslik A., Male C. (2011). Anticoagulation in children undergoing cardiac surgery. Semin. Thromb. Hemost..

[25] Corno A.F., LaPar D.J., Li W., Salazar J.D. (2021). A narrative review of modern approach and outcomes evaluation in congenital heart defects. Transl. Pediatr..

[26] Ohye R.G., Sleeper L.A., Mahony L., Newburger J.W., Pearson G.D., Lu M., Goldberg C.S., Tabbutt S., Frommelt P.C., Ghanayem N.S. (2010). Comparison of shunt types in the Norwood procedure for single-ventricle lesions. Results from the Single Ventricle Reconstruction Trial. N. Engl. J. Med..

[27] Ramachandran P., Anderson J.B., Bove E.L., Hirsch-Romano J.C., Ohye R.G. (2017). Thrombotic complications after stage I palliation for hypoplastic left heart syndrome. J. Thorac. Cardiovasc. Surg..

[28] Manlhiot C., Brandão L.R., Schwartz S.M., Sivarajan V.B., Williams S., Collins T.H., McCrindle B.W. (2011). Management and outcomes of thrombosis in children after congenital heart surgery. J. Thorac. Cardiovasc. Surg..

